# TAM: A method for enrichment and depletion analysis of a microRNA category in a list of microRNAs

**DOI:** 10.1186/1471-2105-11-419

**Published:** 2010-08-09

**Authors:** Ming Lu, Bing Shi, Juan Wang, Qun Cao, Qinghua Cui

**Affiliations:** 1 Department of Biomedical Informatics, Peking University Health Science Center, Beijing, 100191, China; 2 Department of Cardiology, Beijing Military General Hospital, Beijing, 100700, China

## Abstract

**Background:**

MicroRNAs (miRNAs) are a class of important gene regulators. The number of identified miRNAs has been increasing dramatically in recent years. An emerging major challenge is the interpretation of the genome-scale miRNA datasets, including those derived from microarray and deep-sequencing. It is interesting and important to know the common rules or patterns behind a list of miRNAs, (i.e. the deregulated miRNAs resulted from an experiment of miRNA microarray or deep-sequencing).

**Results:**

For the above purpose, this study presents a method and develops a tool (TAM) for annotations of meaningful human miRNAs categories. We first integrated miRNAs into various meaningful categories according to prior knowledge, such as miRNA family, miRNA cluster, miRNA function, miRNA associated diseases, and tissue specificity. Using TAM, given lists of miRNAs can be rapidly annotated and summarized according to the integrated miRNA categorical data. Moreover, given a list of miRNAs, TAM can be used to predict novel related miRNAs. Finally, we confirmed the usefulness and reliability of TAM by applying it to deregulated miRNAs in acute myocardial infarction (AMI) from two independent experiments.

**Conclusion:**

TAM can efficiently identify meaningful categories for given miRNAs. In addition, TAM can be used to identify novel miRNA biomarkers. TAM tool, source codes, and miRNA category data are freely available at http://cmbi.bjmu.edu.cn/tam.

## Background

MicroRNAs (miRNAs) are one class of newly identified important cellular components [1]. At the posttranscriptional level, miRNAs normally act as negative gene regulators by binding to the 3'UTR of target mRNAs through base pairing, which results in the cleavage of target mRNAs or translation inhibition [1]. Increasing evidences suggest that miRNAs play crucial roles in nearly all important biological processes, including cell growth, proliferation, differentiation, development, and apoptosis [2], and that miRNA dysfunctions are associated with various diseases [3]. Since their discovery, the number of identified miRNAs has been increasing dramatically and various high-throughput techniques related to miRNAs are continuously being developed. Microarrays, for example, generate experimental data at rates that exceed knowledge growth. To mine meaningful information of miRNAs, a number of tools and databases have been presented [4-12]. Among these resources, the tools for searching for the gene sets (i.e. KEGG pathways and Gene Ontology) that may be affected by one or multiple miRNAs represent some of the most important tools in miRNA bioinformatics [6,10,11]. A common point of these methods is that they obtain the meaningful gene sets by enrichment analysis of the in-silico predicted miRNA targets. The first limitation of these methods is the high false positives and high false negatives of the predicted miRNA targets [13]. The second limitation of these methods is that they perform analysis based on target genes and only focus on significantly enriched gene sets and therefore may fail to find some functions or biological processes associated with the inputted miRNAs. For example, miR-18a is known to be related to apoptosis [14], but these methods fail to find the pathway "apoptosis" for miR-18a. Finally, it seems difficult for those methods to find novel miRNAs that are related to the inputted miRNAs. Therefore, for a list of miRNAs, for example the upregulated and/or downregulated miRNAs from a miRNA microarray experiment, novel methods are needed to find the patterns behind these miRNAs.

Most of the current tools for miRNA functional annotation are based on predicted miRNA targets, mainly, because of the lack of miRNA knowledge resources. However, functional resources for protein-coding genes are easily available. Therefore, for protein-coding genes, a large number of programs for the annotation of lists of genes have been developed [15] because various gene resources such as the Kyoto Encyclopedia of Genes and Genomes (KEGG) pathway http://www.genome.jp/kegg/ and the Online Mendelian Inheritance in Man (OMIM) compendium http://www.ncbi.nlm.nih.gov/omim/ are available for protein-coding genes. Developing miRNA annotation tools should become more feasible as meaningful miRNA resources are collected. In this study, TAM, a web-accessible program for this purpose is presented. In TAM, miRNAs are integrated into different categories according to the miRNA family, genome locations, functions, associated diseases, and tissue specificity. TAM then evaluates the statistical significance (i.e., overrepresentation or underrepresentation) of each miRNA category among lists of miRNAs using the hypergeometric test. TAM is also able to search for novel miRNAs related to a given list of miRNAs. Finally, we applied TAM to the upregulated miRNAs and downregulated miRNAs in acute myocardial infarction (AMI). As expected, different meaningful miRNA categories have been identified for upregulated and downregulated miRNAs, respectively. This suggested that TAM could be an efficient method and tool for the annotation of meaningful miRNA categories for a list of miRNAs. TAM represents an alternative tool for the processing of outputs of high throughput miRNA experiments.

## Results and Discussion

### miRNA categories

**Figure 1:**
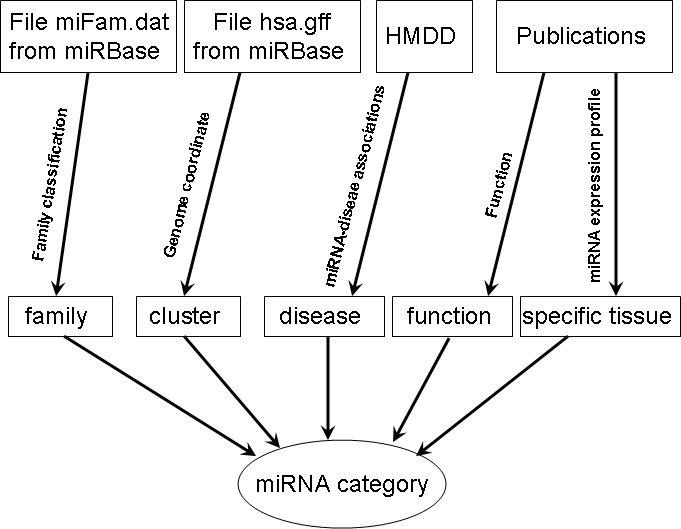
**Classification schemes of miRNA categories**. We integrated miRNAs into various categories according to five classification schemes. They are miRNA family, miRNA cluster, miRNA associated disease, miRNA function, and miRNA tissue specificity. The data sources used to generate the above miRNA categories are also given.

**Figure 2:**
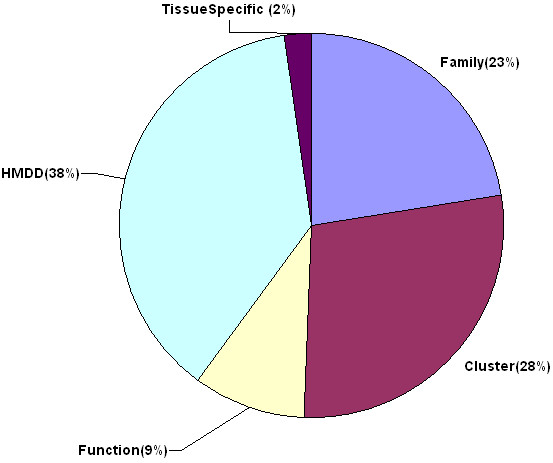
**The distribution of the five types of miRNA categories**. The size of the pie indicates the relative number of miRNA categories in each classification.

In total, we collected 257 miRNA categories according to various classification schemes, such as miRNA family, miRNA cluster, miRNA function, miRNA associated disease, and miRNA tissue specificity (see Materials and Methods). miRNAs that have common characters in any classification scheme will be integrated into one category. Figure 1 shows the detailed flowchart for the miRNA category integration procedure (Figure 1). Among the 257 miRNA categories, 58 belongs to miRNA family category (Family), 72 belongs to miRNA cluster category (Cluster), 24 belongs to miRNA function category (Function), 97 belongs to human miRNA associated disease category (HMDD), and 6 belongs to tissue specificity category (TissueSpecific) (Figure 2). These miRNA categories include more than 400 distinct miRNAs.

### The procedure of TAM analysis

**Figure 3:**
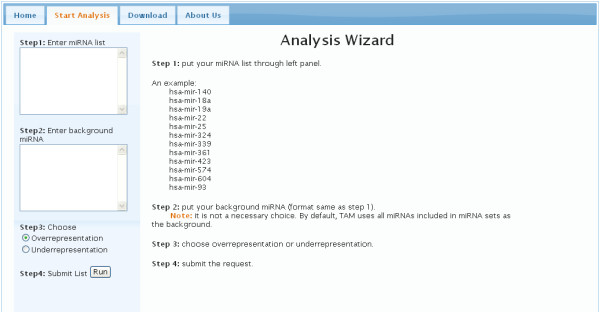
**Analysis flowchart of TAM**.

TAM works in four steps, as shown in Figure 3. In Step 1, a given list of miRNA for analysis is entered. In Step 2, another list of miRNA is entered as background. This step is optional; if a background list is not provided, TAM will use all miRNAs included in the miRNA database as the default background list. In Step 3, the user indicates what analysis (overrepresentation or underrepresentation) is to be performed: overrepresentation or underrepresentation. In Step 4, a result page is generated after the data is submitted. TAM evaluates the significance of each miRNA category for the given miRNAs. The miRNA categories are clustered into five classes including miRNA family, miRNA cluster, miRNA function, miRNA associated disease, and miRNA tissue specificity (Table 1). In the result page, the miRNA category, number of input miRNAs matched this category, percentage of matched miRNAs, fold of the overpresentation or underrepresentation, p value, Bonferroni value, and FDR value are listed, respectively. Other related miRNAs with the given miRNAs in one miRNA category will be shown when the mouse move to corresponding miRNA category.

**Table 1:** Options provided by the TAM tool

Annotation	Description
miRNA family	miRNA categories integrated according to miRNA conservation
miRNA cluster	miRNA categories integrated according to miRNA genome location
miRNA function	miRNA categories integrated according to their function
miRNA associated disease	miRNA categories integrated according to the associated disease
miRNA tissue specificity	miRNA categories integrated according to their tissue specificity

### The upregulated and downregulated miRNAs in acute myocardial infarction (AMI) show different TAM annotations

**Figure 4:**
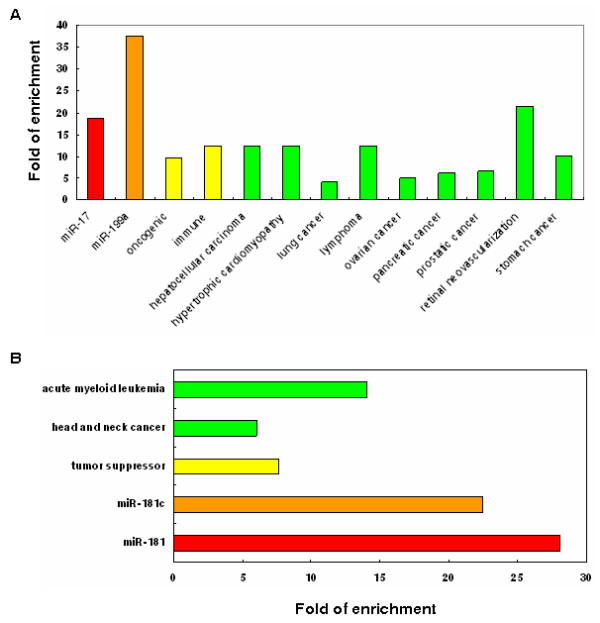
**Enriched miRNA categories for the upregulated miRNAs (A) and the downregulated miRNAs (B) in acute myocardial infarction**. Red, orange, yellow, and green colors represent that the corresponding miRNA category is miRNA family, miRNA cluster, miRNA function, and HMDD miRNA associated disease, respectively.

We first applied TAM to the 16 deregulated miRNA genes from a miRNA microarray experiment (Table 2), in which we previously identified 16 deregulated miRNAs (8 are upregulated in AMI and 8 are downregulated in AMI) in the myocardium tissue of rats with AMI and normal rats [16]. This dataset includes miRNA expression profiles across four time points (the control, 3 day, 7 day, and 14 day), each time point has three samples and each sample has two replicates. In order to investigate the meaningful rules behind these deregulated miRNAs, we identified the enriched miRNA categories for the upregulated miRNAs and downregulated miRNAs, respectively. As a result, the upregulated miRNAs and downregulated miRNAs show obviously different and even opposite enriched miRNA categories. Figure 4 shows the fold of enrichment for the most enriched miRNA categories (P < 0.01). Significantly, the upregulated miRNAs are enriched in miR-199a cluster (P = 1.49 × 10^-4^), whereas the downregulated miRNAs are enriched in miR-181c cluster (P = 2.71 × 10^-3^). For the miRNA family, the upregulated miRNAs and downregulated miRNAs are enriched in miR-17 family (P = 4.03 × 10^-3^) and miR-181 family (P = 1.64 × 10^-3^), respectively. For the miRNA function, the two lists of miRNAs show opposite functions. The upregulated miRNAs are enriched in oncogenic function (P = 2.56 × 10^-4^) and immune system function (P = 1.01 × 10^-3^), whereas the downregulated miRNAs are enriched in tumor suppressor function (P = 1.36 × 10^-4^). Consequently, both lists of miRNAs are enriched in tumors (Table 2). In addition, the upregulated miRNAs are enriched in hypertrophic cardiomyopathy and atrophic muscular disorders, whereas the downregulated miRNAs are enriched in cardiac arrhythmias, cardiomegaly, coronary artery disease, and polycythemia vera (Table 2). In function, the upregulated miRNAs are also enriched in Akt pathway, cell cycle, HIV latency, hormones regulation, stem cell regulation, immune, and inflammation; the downregulated miRNAs are also enriched in cardiogenesis, hormones regulation, and muscle development. Finally, although not so significant, the downregulated miRNAs tend to be enriched in function of muscle development (P = 0.01) and tend to be heart and muscle specific (P = 0.15). The enriched miRNA categories of AMI upregulated and downregulated miRNAs might provide help in understanding AMI. For example, the upregulated miRNAs are enriched in function of oncogenes, whereas the downregulated miRNAs are enriched in function of tumor suppressors. This result suggests that the deregulated miRNAs tend to stimulate the proliferation of cardiac fibroblasts, which is further helpful for collagen synthesis and cardiac remodeling. This may be a compensatory mechanism for acutely infracted myocardium.

**Table 2:** Significant miRNA categories of upregulated and downregulated miRNAs in AMI obtained by TAM

miRNA category	Upregulated miRNAs	Downregulated miRNAs
	(miR-31; miR-18a;miR-18b; miR-214;miR-223;miR-923; miR-711;miR-199a)	(miR-499;miR-29b;miR-126; miR-1;miR-181d;miR-181c; miR-451;miR-26b)

**Family**	miR-17;miR-199	miR-181;miR-26

**Cluster**	miR-199a	miR-1;miR-144;miR-181c; miR-29a;miR-29b

**Function**	Akt pathway;cell cycle; HIV latency;Hormones regulation; stem cell regulation;immune; inflammation;oncogenic	Cardiogenesis; Hormones regulation; tumor suppressor; Muscle development

**HMDD**	Cancer; hypertrophic cardiomyopathy; atrophic muscular disorders;	Cancer; Cardiac Arrhythmias; Cardiomegaly; Coronary Artery Disease; polycythemia vera;

**Tissue Specific**	Not available	Not available

To valid our method, we applied TAM to the deregulated miRNAs of AMI from another independent miRNA expression profiling experiment of AMI rat model by Rooij et al.[17]. In their study, Rooij et al. identified 39 upregulated miRNAs and 46 downregulated miRNAs, respectively. As a result, although the deregulated miRNAs from Rooij et al.' experiment seem quite different from those of Shi et al.', the enriched miRNA categories identified by TAM have a good consistency across these two independent experiments. For example, the upregulated miRNAs from Rooij et al.' experiment are also enriched in miR-199a cluster (P = 4.33 × 10^-3^), miR-199 family (P = 4.33 × 10^-3^), cell cycle (P = 6.37 × 10^-3^), stem cell regulation (P = 1.82 × 10^-6^), inflammation (P = 3.14 × 10^-3^), and onco-miRNAs (P = 5.73 × 10^-5^). For HMDD category, the upregulated miRNAs are also enriched in various cancer, hypertrophic cardiomyopathy (P = 0.04) and atrophic muscular disorders (P = 4.54 × 10^-12^); the downregulated miRNAs from Rooij et al.' experiment are also enriched in miR-29a cluster (P = 7.37 × 10^-3^), miR-29b cluster (P = 7.37 × 10^-3^), hormones regulation (P = 2.14 × 10^-7^), miRNA tumor suppressor (P = 9.23 × 10^-3^). For HMDD category, the downregulated miRNAs are also enriched in various cancer, and polycythemia vera (P = 7.01 × 10^-3^).

### Prediction of novel miRNAs related to AMI

As discussed previously, one of the limitations of target-based pathway enrichment analysis of miRNAs is that it can not predict novel miRNAs related to the inputted miRNAs. For TAM, it is very easy to perform this kind of analysis because TAM integrated miRNAs directly but not integrated miRNAs through miRNA targets. In the enriched miRNA category, the other miRNAs that are not included in the input miRNA list could be potential novel miRNAs related to the inputted miRNAs. For example, TAM analysis showed that the 16 deregulated miRNAs in AMI from Shi et al.'s study are enriched in the function of muscle development (P = 0.04). Among the 11 miRNAs in this category, two (miR-1 and miR-499) are included in the 16 inputted miRNAs. The other 9 miRNAs (miR-24, miR-124, miR-133a, miR-23a, miR-133b, miR-206, miR-221, miR-222, and miR-208b) in this category are predicted to be potential novel AMI related miRNAs. We confirmed four (miR-24, miR-133a, miR-221, and miR-222) of the nine miRNAs (44.4%) are related to AMI based on the deregulated miRNAs from another independent study by Rooij et al.[17]. The results indicate that TAM is a highly reliable tool for predicting novel miRNAs that are related to inputted miRNAs.

## Discussion

As the rapid development of high-throughput biological techniques, it is increasingly important to mine meaningful patterns for a given list of miRNAs. As described above, TAM represents one important tool for this purpose. Unlike tools based on in-silico predicted miRNA targets, TAM integrated miRNAs into groups directly based on miRNA annotations. Therefore, TAM represents a new class of methods for the above purpose and represents an alternative tool for the annotations of a given list of miRNAs. Furthermore, TAM is able to predict novel miRNAs that are related to the inputted miRNAs. This enables users to find novel miRNA biomarkers for their experiments. In addition, TAM is highly dependent on the data of integrated miRNA sets and will be improved greatly when more miRNA annotation data becomes available in the future.

## Conclusions

In this study, we presented a method to identify overrepresented and/or underrepresented miRNA categories for a given list of miRNAs. Moreover, an online tool, TAM, for annotations of human miRNAs based on various miRNA sets is developed. After applying TAM to deregulated miRNAs in AMI, we show that the upregulated miRNAs and the downregulated miRNAs in AMI are enriched in different and even opposite miRNA categories, which is helpful for the understanding of AMI. In addition, TAM can be used to predict novel miRNAs that are mostly related to the input miRNAs. TAM is useful for providing potential clues for miRNAs of interest. Furthermore, TAM is scalable and will grow and improve as more miRNA resources become available. In addition, TAM can be easily reconfigured for use with other species.

## Methods

### miRNA sets

miRNA sets are defined as groups of miRNAs that have meaningful relationships. If any two miRNAs have meaningful relationships, for example they are associated with the same diseases, they are then integrated into one miRNA set. Here, miRNA sets were collected according to miRNA family, genome locations, function, associated diseases, and tissue specificity. Studies have indicated that miRNAs in one family are most likely derived from duplications of common ancestor miRNAs [18,19], and tend to act together in various functional processes [20,21]. Therefore, miRNAs in one family can be considered as one miRNA set. The miRNA family data from the miRBase database was downloaded [7] and utilized in this study.

miRNAs are not located randomly in the genome but tend to exist in clusters [22]. MiRNAs in a cluster are likely to be co-transcribed and have similar expression patterns [23]. Therefore, these clustered miRNAs may be involved in similar biological processes. In this study, miRNA clusters were identified by grouping miRNAs that were within a distance of 50 kb in the chromosomes, according to the observation of Baskerville and Bartel [23]. The integrated miRNAs were also manually integrated into different sets according to their functions, as reported in publications. For example, miRNAs that were associated with the immune system were collected from a recent review paper published in Cell [24]. The miRNA sets were generated by miRNA-associated diseases based on the Human MicroRNA Disease Database (HMDD, http://cmbi.bjmu.edu.cn/hmdd), a database for miRNA disease associations [3]. The tissue-specific index values of miRNA were obtained from the study of Lu et al.[3], and tissue-specific miRNA sets were generated by collecting miRNAs with tissue specificity index values of greater than or equal to 0.7. Finally, according to the methods described above, 257 miRNA sets were generated. These miRNA sets are available for download at the TAM website.

### Evaluation of statistical significance

The hypergeometric test [25], was used to determine the significant overrepresentation and/or underrepresentation of the miRNA sets among a list of miRNAs of interest. Assuming that *P *represents the number of miRNAs included in all miRNA sets, *S *represents the number of miRNAs included in miRNA set *A*, *HP *represents the number of input miRNAs included in *P*, and *HS *represents the number of miRNAs that are of interest included in *S*, the probability of *HS *miRNAs of interest in miRNA set *A *is

(1) $$P(x=HS)=\frac{C_{HP}^{HS}\times C_{P-HP}^{S-HS}}{C_{P}^{S}}$$

where the symbol "C" means the combination operation. Therefore, the statistical significance of miRNA set *A *among the miRNAs of interest are represented by Formula (2) and (3):

(2) $$P(overrepresentation)=\sum_{h=HS}^{S}P(x=h)$$

(3) $$P(underrepresentation)=\sum_{h=0}^{HS}P(x=h)$$

Finally, the P values for all miRNA sets are adjusted by *Bonferroni *and *FDR *corrections.

## Availability and requirements

Project name: TAM.

Project home page: http://cmbi.bjmu.edu.cn/tam.

Operating system: Platform independent.

Programming language: Python.

Other requirements: Apache 1.22, Jquery, Extjs, and Django.

License: GPL v3.

## Authors' contributions

QC designed this study and wrote the manuscript. ML implemented the algorithms and created the web server. BS and ML analyzed the deregulated miRNAs of AMI. JW and QC curated the HMDD miRNA categories. All authors have read and approved the final manuscript.
